# P33 Metagenomic sequencing of sputum enables accurate, non-invasive diagnosis of community-acquired pneumonia

**DOI:** 10.1093/jacamr/dlaf230.040

**Published:** 2025-12-04

**Authors:** Mahboobeh Behruznia, Nicola Cumly, Samuel Quarton, Kirsty McGee, David Thickett, Dhruv Parekh, Elizabeth Sapey, Alan McNally

**Affiliations:** Microbes, Infections and Microbiomes, University of Birmingham, Edgbaston, Birmingham, B15 2TT, UK; Microbes, Infections and Microbiomes, University of Birmingham, Edgbaston, Birmingham, B15 2TT, UK; Department of Inflammation and Ageing, University of Birmingham, Edgbaston, Birmingham, B15 2TT, UK; Department of Inflammation and Ageing, University of Birmingham, Edgbaston, Birmingham, B15 2TT, UK; Department of Inflammation and Ageing, University of Birmingham, Edgbaston, Birmingham, B15 2TT, UK; Department of Inflammation and Ageing, University of Birmingham, Edgbaston, Birmingham, B15 2TT, UK; Department of Inflammation and Ageing, University of Birmingham, Edgbaston, Birmingham, B15 2TT, UK; Microbes, Infections and Microbiomes, University of Birmingham, Edgbaston, Birmingham, B15 2TT, UK

## Abstract

**Background:**

Community-acquired pneumonia (CAP) remains a major cause of hospital admission and mortality, yet the causative pathogen is rarely identified. Conventional diagnostics, including sputum culture, blood culture and multiplex PCR, lack sensitivity, breadth, or speed. Consequently, most patients receive empirical antibiotics, with implications for antimicrobial stewardship and patient outcomes. Invasive sampling (e.g. bronchoalveolar lavage) improves diagnostic yield but is often impractical in routine care. Nanopore metagenomic sequencing offers unbiased, real-time pathogen detection, but its utility using non-invasive specimens such as swabs and sputum remains underexplored.

**Methods:**

We performed Nanopore-based metagenomic sequencing on 60 respiratory samples (46 nasopharyngeal swabs, 12 sputa, 2 pleural fluids) from 38 adults hospitalized with CAP and 8 matched controls. The higher number of swabs reflects the practical challenges of sputum collection in hospitalized CAP patients, where non-production is common. We also performed a paired analysis on patients who had both swab and sputum collected, enabling direct comparison of sample types. Protocols incorporated host DNA depletion, microbial and viral workflows, and rigorous bioinformatics (taxonomic classification, genome breadth and read dispersion metrics) to distinguish true pathogens from commensals. Sequencing results were compared with routine microbiological diagnostics and reviewed in a multidisciplinary clinical context.

**Results:**

Host depletion reduced human DNA contamination and enriched microbial reads. Sputum consistently provided higher bacterial read counts (median 192 426 versus 14 025 per sample), deeper genome coverage and more uniform read distribution. Clinically relevant pathogens identified in sputum included *Streptococcus pneumoniae, Streptococcus pyogenes, Moraxella catarrhalis and Mycoplasmoides pneumoniae*. Two samples (one sputum and one swab) from a patient were confirmed as *M. pneumoniae* positive by qPCR but missed by routine diagnostic tests. Within patient comparison with paired swab-sputum samples showed that swabs were dominated by upper airway commensals (e.g. *Dolosigranulum pigrum*), whereas sputum reliably reflected lower respiratory tract infections. Metagenomics additionally identified polymicrobial infections and potential resistance determinants in sputum and pleural fluid samples.

**Conclusions:**

Our study demonstrates that sputum-based Nanopore metagenomic sequencing is a powerful, non-invasive alternative to invasive lower respiratory tract sampling in CAP. It improves pathogen identification and expands coverage beyond culture and PCR assays. By bridging laboratory workflows with clinical decision-making, this approach could improve CAP management—particularly for patients able to provide sputum —by supporting rapid targeted therapy, optimizing antibiotic use and reducing diagnostic uncertainty in one of the most common serious infections worldwide.

**Impact:**

This work highlights the tangible clinical–laboratory connection: how metagenomic sequencing, applied to a readily available specimen, can shift pneumonia diagnostics from empiricism toward precision. The findings position sputum metagenomics as a scalable tool for hospitals, especially in settings where invasive sampling is not feasible, with direct relevance to antimicrobial stewardship programmes and patient outcomes.

